# Heterotrophic bacterial bioleaching and sequestration of uranium from mineral resources: a review

**DOI:** 10.3389/fmicb.2026.1803404

**Published:** 2026-04-21

**Authors:** Tariq M. Bhatti, Olli H. Tuovinen

**Affiliations:** 1 Pakistan Institute of Engineering and Applied Sciences, Islamabad, Pakistan; 2 Department of Microbiology, Ohio State University, Columbus, OH, United States

**Keywords:** biosorption, EPS, organic acids, siderophores, uranium bioleaching, uranium phosphates

## Abstract

Heterotrophic bacterial leaching of uranium from rocks and ores and cellular sequestration are examined in this review. Heterotrophic microbial bioleaching solubilizes uranium by acidolysis and complexolysis and involves sequestration by organic acids, extracellular polymeric substances (EPS), lipopolysaccharides, and siderophores produced by bacteria. Citric and oxalic acids are often the dominant organic acids, but spent growth media also contain mixtures of other < C6 carboxylic acids. The leach solution (lixiviant) is based on organic acids in spent media, and the mode of leaching is proton attack on uranium ore coupled with sequestration of uranyl ions (${\text{UO}}_{2}^{2+}$) by organic acids, thus preventing precipitation in the lixiviant. Many heterotrophs in different bacterial genera have been tested for uranium bioleaching and sequestration from mineral resources, and some notable examples include actinomycetes, *Bacillus* spp., and *Pseudomonas* spp. Commercial applications of heterotrophic bacterial bioleaching and biomass sorption of uranium have not emerged. Uranium sequestration in biomass constituents such as EPS and siderophores can represent a significant fraction of biomass sorption and make uranyl ions biologically unavailable to the cells. Precipitation with phosphates particularly immobilizes uranyl ions and can result in crystallized mineral phases. The biological reduction of U(VI) to solid-phase U(IV) is an immobilization technique to prevent uranium migration in aquifers and reduce environmental impact, potentially as part of remediation strategies such as permeable reactive barrier construction.

## 1 Introduction

Many heterotrophic bacteria, as well as fungi, can solubilize uranium from non-sulfide mineral resources primarily due to dissolution and sequestration by carboxylic acids and siderophores. Uranium sequestration involves carboxyl, amino, hydroxyl, phosphate, and sulfhydryl functional groups in extracellular polymeric substances (EPS), siderophores, and lipopolysaccharides (LPS, only in Gram-negative bacteria) of the cell wall. The cell envelopes of Gram-positive and Gram-negative bacteria possess an electronegative charge due to the presence of carboxyl, amino, hydroxyl, phosphate, and sulfhydryl functional groups in the cell wall, enabling them to attract positively charged metal ions to the surface binding sites (Kulczycki et al., 2002, 2010; Slavin et al., 2017; Draviana et al., 2025). Thus, the oxidized form of dissolved uranium, ${\text{UO}}_{2}^{2+}$, is reactive and binds to cell wall functional groups (Hufton et al., 2021; Krawczy-Bärsch et al., 2022; Hoyle-Gardner et al., 2023). The physicochemical interaction of bacteria with uranium is governed by intermolecular forces between functional groups on the cell wall surface (Merroun et al., 2003; Ojeda et al., 2008a,b, 2009).

Uranium mineralization in rocks and ores may be associated with rare earth elements (REEs) (Reynier et al., 2021; Brown et al., 2023), which can also dissolve in contact with heterotrophic bacteria. REEs are of special interest because this group is in short supply in many critical areas of technology (Golzar-Ahmadi et al., 2024). Many potential bioleaching applications of heterotrophic bacteria have been explored over the years, but to date, none have been commercialized. These include biologically produced organic acid solutions contacted with lateritic and sulfide ores and tailings, as well as spent batteries, waste printed circuit boards, municipal solid waste, and other urban waste streams, coal fly ash, and industrial sludges (Li et al., 2015; Dusengemungu et al., 2021; Ji et al., 2022; Magoda and Mekuto, 2022; Adetunji et al., 2023; Kumar et al., 2026).

Factors affecting the heterotrophic bacterial leaching and sequestration of uranium include growth and culture parameters: temperature, pH, aeration, carbon source and other nutrients, solution chemistry, and metabolic pathways. Many physical and chemical factors are comparable in the heterotrophic bioleaching and acid ferric-iron bioleaching of uranium from ores and rocks, such as particle size, pulp density, contact time, chemical composition and mineralogy of the ore sample, acid demand, secondary dissolution products, and solid-phase transformations (Kaksonen et al., 2020; Bhatti and Tuovinen, 2025). These factors have been discussed in the literature repeatedly. *In situ* recovery mining of uranium is practiced with acid solutions injected in the subsurface. Microbial communities in these situations have abundant aerobic and anaerobic heterotrophic bacteria, which participate in uranium reduction, oxidation, precipitation, and sequestration (Zammit et al., 2014; Islam and Sar, 2016; Jroundi et al., 2020). Uranium in these roll-front mineralizations is usually a mixture of crystallized U(IV) in coffinite [U(SiO_4_)·nH_2_O] and uraninite (UO_2_), but the deposits also contain a variable fraction of a non-crystallized phase of U(IV). This reduced, non-crystallized phase of uranium is of biogenic origin (Bhattacharyya et al., 2017). Thus, the activities of these heterotrophs contribute to redox transformations and solubilities of uranium.

Uranium contamination in the ambient environment is a point-source pollution problem, especially from active and former uranium mills and mine sites, as well as waste from various uranium-processing industries. On a global scale, elevated uranium levels in groundwater have been reported in some regions of the world, posing serious human health hazards in drinking water. This non-point-source uranium pollution in groundwater is the result of fluctuating geochemical and mineralogical conditions, spanning from reduced to intermediate oxic and fully oxidized conditions depending on clays and lateritic layers overlaying granitic aquifers (Mitra et al., 2025). Contamination in subsurface sediments and groundwater aquifers is long term and persistent. Geochemical and biological control of uranium mobility is contingent upon uranium solution and solid-phase chemistry and is intertwined with microbial redox and phase transformations, which impact uranium speciation, solubility, bioaccessibility, bioavailability, and toxicity (Merroun and Selenska-Pobell, 2008; Belli and Taillefert, 2017; Majumder and Wall, 2017; Kolhe et al., 2018; You et al., 2021).

This narrative review examines research advances on the dissolution and sequestration of uranium from low-grade ores, tailings residues, mining wastes, rocks, and cut-off grade ores by heterotrophic bacteria. Relevant information published since 2000 was searched using the internet and Google Scholar, Web of Science, ScienceDirect, recent papers, publications in special topic archives in scientific journals, various conference volumes, and book chapters. All mineral formulas in this review are normalized per http://www.mindat.org. The studies in this field of bioleaching have addressed the dissolution of uranium by organic acids and sequestration by siderophores and EPS. The bioleaching, bioprecipitation, biosequestration, and bioreduction of uranium are major biotransformations involved in the bioremediation of uranium-contaminated soils and mine sites (Lloyd and Macaskie, 2000; Finneran et al., 2002; Gao and Francis, 2008; Liu et al., 2015; Lakaniemi et al., 2019; Akash et al., 2022; Yu and Cui, 2023; Wufuer et al., 2025). The biological context of N, S, Fe, and Mn cycles and concepts of bioremediation in uranium-impacted environments have been expertly discussed in the past (Mkandawire, 2013; Williams et al., 2013; Newsome et al., 2014; Campbell et al., 2015; Safonov et al., 2021; Kaplan et al., 2024; Wei et al., 2024). Chemical mitigation techniques have also been discussed in the review literature (Gavrilescu et al., 2009; Banala et al., 2021b; Ighalo et al., 2024; Jindal and Patni, 2026; Lin et al., 2026). Numerous publications cited in this review have presented phase and stability diagrams, schematics of biological sequestration, and chemical equations of redox and phase transitions. This narrative review focuses on individual studies to examine the multitude of experimental approaches, heterotrophic bacterial diversity, analytical aspects, and the state of the art to date.

## 2 Carboxylic acid production by heterotrophic bacteria

The redox speciation of uranium greatly controls its mobility and solubility in the environment. Uranium in contaminated sediments and aquifers exists as reduced U(IV), which is immobile and a solid or colloidal phase, and oxidized U(VI) as the uranyl ion (${\text{UO}}_{2}^{2+}$), a thermodynamically stable species. Variables affecting uranium redox transformations by microorganisms include pH, redox potential, other metal ions, and cellular and extracellular ligands and their stabilities and affinities. The pH is a significant environmental factor in the speciation of uranium because solubility, sequestration, and precipitation are all sensitive to solution pH. Carboxylic acids form soluble complexes with ${\text{UO}}_{2}^{2+}$. For example, the pH dependence of ${\text{UO}}_{2}^{2+}$ ion complexation by citric acid involves three major ${\text{UO}}_{2}^{2+}$-citrate complexes with progressively increasing U=O bond lengths over a pH range of 2.0–9.5 (Pacilis and Pemberton, 2003). The [(UO_2_)_2_Cit_2_]^2−^ complex is the first predominant species of ${\text{UO}}_{2}^{2+}$ ion, which exists at pH 3.0–5.0. At pH >6.5, the [(UO_2_)_2_Cit_2_]^2−^ complex undergoes an interconversion to form [(UO_2_)_3_Cit_3_]^3−^ and (UO_2_)_3_Cit_2_ complexes (Pacilis and Pemberton, 2003). The formation of soluble uranyl complexes with carboxylic acids provides a means of recovering uranium from mineral resources.

Thus, organic acids provide protons (H^+^) and ligands that dissolve uranium and metals (Wen et al., 2024; Lisińska et al., 2022), and organic acid production is of key importance in the bioleaching by heterotrophic bacteria (Li et al., 2021). Short-chain (≤C6) volatile fatty acids are common metabolites in heterotrophic metabolism and fermentation and include acetic (C_2_H_4_O_2_), citric (C_6_H_8_O_7_), gluconic (C_6_H_12_O_7_), formic (CH_2_O_2_), lactic (C_3_H_6_O_3_), malic (C_4_H_6_O_5_), malonic (C_3_H_4_O_4_), oxalic (C_2_H_2_O_4_), pyruvic (C_3_H_4_O_3_), and succinic (C_4_H_6_O_4_) acids. They can promote acid attack at a pH range of 1–4 as well as sequester dissolved uranyl and other metal ions at higher pH values, alleviating metal precipitation in the leach solution. Heterotrophic bacteria produce carboxylic acids from carbohydrates by fermentative metabolism, especially under anaerobic conditions. Bacteria excrete acids outside the cells to avoid their inhibition (Jarboe et al., 2013). Many bacteria are facultative anaerobes, fermenting sugars in the absence of oxygen and switching to respiratory metabolism under aerobic conditions. Glucose as the carbon and energy source is utilized by the glycolytic pathway (Embden–Meyerhof–Parnas pathway) and is converted to two equivalents of pyruvic acid. Pyruvic acid is also formed in the metabolism of other sugars and carbohydrates. A brief synopsis of the relevant pathways has been presented by Soto-Montandon et al. (2025).

The major pathways downstream from pyruvate vary in metabolites and depend on the bacterial genus and species (Murali et al., 2017; Behera et al., 2021; Tomás Pejó et al., 2023). Key metabolic routes are the tricarboxylic acid (TCA) cycle, the pentose phosphate pathway, and the glyoxylate cycle. Many heterotrophic bacteria are known to produce citric acid at much higher levels than needed in the TCA cycle. Homofermentative citric acid production by the filamentous fungus *Aspergillus niger* is among the best-known examples because it has been used for decades for commercial production of citric acid as a feedstock in the pharmaceutical and food industries (Latif et al., 2025). The pentose phosphate pathway is parallel to the glycolytic pathway and primarily serves an anabolic function, but some metabolites can link back to glycolysis. In some bacteria, the TCA cycle is not complete because of the lack or low activity of one of the key enzymes. In those instances, the pentose phosphate pathway is the dominant catabolic pathway of glucose metabolism.

Lactic acid (C_3_H_6_O_3_) is formed from glucose either via the homofermentative or heterofermentative pathway. Acetic acid, with glucose as the substrate, is produced by the oxidation of acetaldehyde, which is first produced by pyruvate decarboxylation. Another route to acetic acid is via ethanol oxidation to acetaldehyde, the precursor. Mixed acid fermentation varies in the products of carboxylic acids. The glyoxylate cycle uses two-carbon compounds (e.g., acetate, acetaldehyde) and is a variant of the TCA cycle (Yang et al., 2022). It centers on the conversion of acetyl-CoA to succinate as a precursor to the synthesis of cellular carbohydrates.

## 3 Case studies of the bioleaching of uranium by heterotrophic bacteria

Hewedy et al. (2013) tested nine actinomycete isolates for uranium bioleaching of a sample (Ø ≤ 2 mm) of low-grade ferruginous sandstone ore, which contained 0.032% U, 0.0237% Th, and 0.217% ΣREEs. The study involved shake flask experiments with a 1% ore sample in distilled water, pH 7.0, at 30 °C for 48 h. The highest uranium extraction of 13% U was achieved with a *Streptomyces aureofaciens* isolate, and several other *Streptomyces* cultures yielded values in the range of 4–13% U. The mode of uranium dissolution is not clear because no carbon source was added for organic acid production by the test culture. The REE dissolution from the same sandstone sample was in the range of 12%−37%. The ore sample was from Wadi Abu Thor, southwestern Sinai (Egypt), and was mainly composed of sand and silt grains embedded in a clay matrix that contained 1.15% organic matter. Hewedy et al. (2013) speculated that *S. aureofaciens* could use the organic fraction of the sample as a carbon substrate, but this was not substantiated by data. Geologically buried organic matter is recalcitrant and not readily biodegradable within such a short time frame. The results for the bioleaching are equivocal because the pH and acid production were not monitored, and abiotic leaching was not tested.

Abdulla et al. (2018) tested 11 actinomycete isolates for uranium bioleaching of a sample of low-grade sandstone (0.022% U), manganese ore (0.055% U), and granite (0.077% U) in shake flask leaching experiments using 2%−10% wt/vol pulp density (−60 mesh = −250 μm) at pH 6.8–7.0 at 28 °C. The maximum uranium extraction from the samples in shake flask cultures was achieved with a 4% pulp density and 10 days of contact time with *Streptomyces* isolates. The leaching solution was a culture medium with mineral salts and 10 g L^−1^ glucose. Organic acids were not analyzed in leach solution samples during the 12-day time course. An isolate of *Streptomyces bacillaris* solubilized 38% U (44% U_3_O_8_) from the low-grade sandstone sample that contained the secondary uranium mineral jachymovite [(UO_2_)_8_(SO_4_)(OH)_14_·13H_2_O] (Table 1). Another *Streptomyces* isolate solubilized 33% U (38.6% U_3_O_8_) from the manganese ore sample, and a third *Streptomyces* isolate solubilized 16.8% U (17% U_3_O_8_) from the granite sample. The extent of solubilization was greater in samples that were sterilized before culture inoculation. The method of sterilization was not stated by Abdulla et al. (2018); however, if sterilization was performed by autoclaving, heating at 120 °C for 20 min would enhance chemical dissolution.

**Table 1:** Glossary of uranium minerals and other solid phases mentioned in the text.

Name	Ideal formula
Ankoleite	K_2_(UO_2_)_2_(PO_4_)_2_·6H_2_O
Autunite	Ca(UO_2_)_2_(PO_4_)_2_·10-12H_2_O
Bassetite	Fe^2+^(UO_2_)_2_(PO_4_)_2_·10H_2_O
Britholite	(Ce,Ca,Th,La,Nd)_5_(SiO_4_,PO_4_)_3_(OH,F)
Calcium phosphate	Ca_3_(PO_4_)_2_
Chernikovite	(H_3_O)_2_(UO_2_)_2_(PO_4_)_2_·6H_2_O
Coffinite	U(SiO_4_)·nH_2_O
Hydroxyapatite	Ca_5_(PO_4_)_3_OH
Jachymovite	(UO_2_)_8_(SO_4_)(OH)_14_·13H_2_O
Mackinawite	FeS
Magnetite	Fe^2+^${\text{Fe}}_{2}^{3+}$O_4_
Meta-autunite	Ca(UO_2_)_2_(PO_4_)_2_·6H_2_O
Metanatroautunite	NaUO_2_PO_4_·3H_2_O
Metavanmeersscheite	U^6+^(UO_2_)_3_(PO_4_)_2_(OH)_6_·2H_2_O
Monazite	(Ce,La,Nd,Th)PO_4_
Ningyoite	(U,Ca,Ce)_2_(PO_4_)_2_·1-2H_2_O
Pitchblende	(U^4+^U^6+^)O_2_
Pyrite	FeS_2_
Pyrrhotite	Fe_n_S_n+1_, n < 1
Saléeite	Mg(UO_2_)_2_(PO_4_)_2_·10H_2_O
Uramphite	NH_4_UO_2_PO_4_·6H_2_O
Uraninite	UO_2_
Uranium oxide phosphate	U_2_O(PO_4_)_2_
Uranium(IV) phosphate	U_3_(PO_4_)_4_
Uranyl metaphosphate	UO_2_(PO_3_)_2_
Uranyl phosphate	UO_2_(PO_4_)_2_
Uranyl phosphate hydrate	(UO_2_)_3_(PO_4_)_2_·H_2_O
Uranyl phosphate tetrahydrate	(UO_2_)_3_(PO_4_)_2_·4H_2_O
Uranyl hydrogen phosphate	UO_2_HPO_4_·4H_2_O
Uvanite	U_2_V_6_O_21_·15H_2_O
Vanmeersscheite	U^6+^(UO_2_)_3_(PO_4_)_2_(OH)_6_·4H_2_O
Vivianite	${\text{Fe}}_{3}^{2+}$(PO_4_)_2_·8H_2_O

All mineral data have been normalized to formulas at https://www.mindat.org.

Abdulla et al. (2018) also tested static bioleaching with *S. bacillaris* using a truncated cone that contained 1 kg of low-grade sandstone ore (0.022% U). The recirculated leach solution (1 L) was a mixture of mineral salts, 10 g glucose, and 1 g chlorphenesin (a fungal inhibitor). The ore sample (Ø ≤ 2 mm) was pretreated with sulfuric acid to satisfy the acid demand. The bioleach solution contained 12.5 mg U L^−1^ U, equivalent to 5.68% U, after 20 days of contact (Abdulla et al., 2018), which is clearly not an efficient experimental treatment. In a parallel experiment, the sulfuric acid-based leaching solution (28 g H_2_SO_4_ L^−1^) dissolved 9% uranium from the low-grade sandstone sample.

Hussien et al. (2016) tested *Pseudomonas fluorescens* SHA 281 in leaching experiments with sandstone uranium ore (0.030% U) and waste rock (0.017% U) samples. The culture produced gluconic acid (8.4 g L^−1^) in 5 days of incubation. The extent of leaching in inoculated glucose medium reached 58 and 60% U dissolution from the sandstone and waste rock samples, respectively, after 8 days of contact in shake flasks with 10% pulp density at 35 °C and 175 rpm. Hussien et al. (2016) also evaluated, to a limited extent, the effects of pulp density, shaking speed, time course, and temperature on uranium leaching in shake flask experiments.

**Figure 1:**
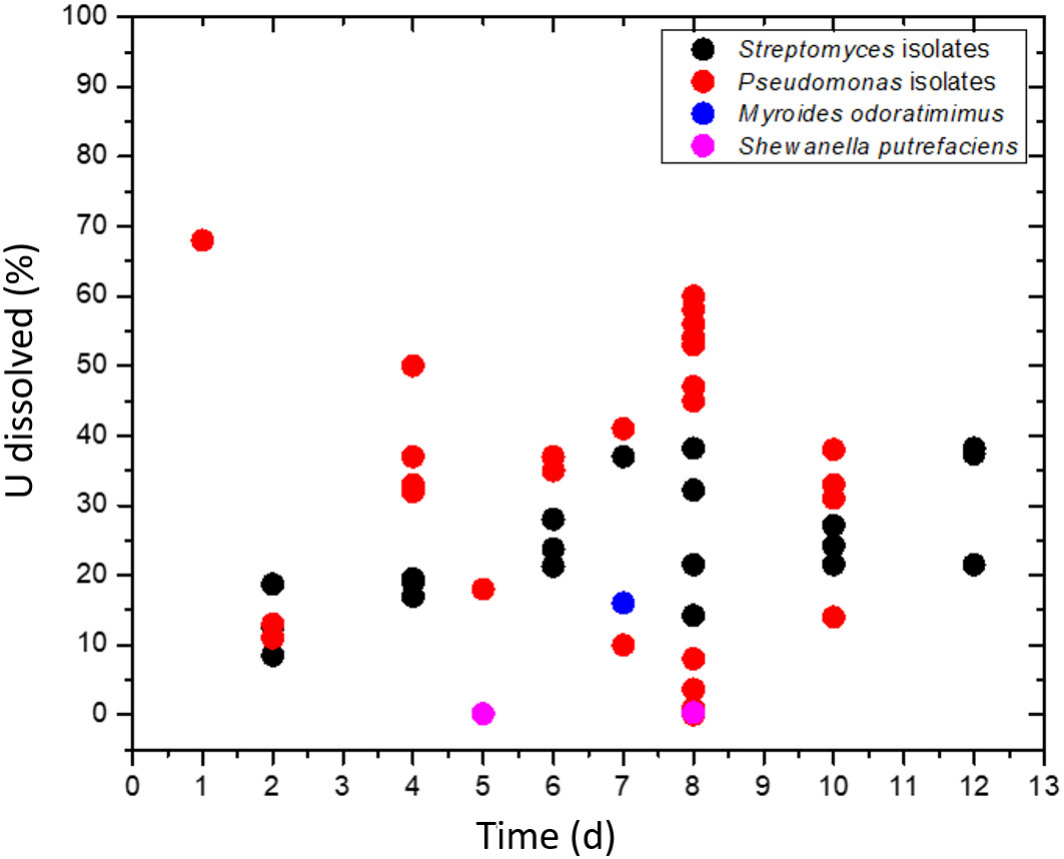
A scatter plot of uranium bioleaching data for *Streptomyces* and *Pseudomonas* isolates, *Myroides odoratimimus*, and *Shewanella putrefaciens*. Data were pooled from Abdulla et al. (2018), Hussien et al. (2016), Desouky et al. (2016), Hewedy et al. (2013), Munir et al. (2019), and Kalinowski et al. (2004, 2006).

Data on the uranium bioleaching by heterotrophic bacteria are summarized in Figure 1 and Table 2, pooled from the available information in the literature. These exploratory studies lack experimental details, sometimes including the absence of abiotic controls, and analytical information on many parameters that impact the outcome of heterotrophic uranium bioleaching. The applicability of heterotrophic bacteria for uranium leaching has not been critically assessed in these studies, perhaps because of the preliminary nature of the experiments. It is apparent that the research has not produced promising results that would warrant detailed follow-up parameter optimization and scale-up studies.

**Table 2:** Summary of the bioleaching of uranium from ores and rocks by heterotrophic bacteria.

Bacteria	Uranium ore	Mineralization	Uranium content	Biolixiviant	Leaching conditions^a^	Uranium recovery	Reference
*Streptomyces aureofaciens*	Ferruginous sandstone ore	Wadi Abu Thor, Sinai, Egypt	0.032% U	Organic acids	Shake flask, 1% PD, pH 7.0, particle size ≤ 2 mm, 30 °C, 150 rpm, 2 days, one-step process	13% U	Hewedy et al. (2013)
*Streptomyces bacillaris* UA12	Sandstone ore	Um Bogma formation, Egypt	0.022% U	Organic acids	Shake flask, 4% PD sterile ore, initial pH 6.9, final pH 4.9, particle size < 250 μm, 28 °C, 150 rpm, 10 days, one-step process	38% U	Abdulla et al. (2018)
Isolate UA5	Manganese ore	Um Bogma formation, Egypt	0.047% U	Organic acids	Shake flask, 4% PD sterile ore, pH 6.8–7.0, initial pH 5.5, final pH 4.0, particle size < 250 μm, 28 °C, 150 rpm, 10 days, one-step process	33% U	Abdulla et al. (2018)
Isolate U7 (uncultured bacterium clone)	Granite rock	Wadi Nysrine, Sinai, Egypt	0.065% U	Organic acids	Shake flask, 4% PD sterile ore, pH 6.8–7.0, initial pH 7.0, final pH 5.1, particle size < 250 μm, 28 °C, 150 rpm, 10 days, one-step process	16.8% U	Abdulla et al. (2018)
*Streptomyces bacillaris* UA12	Sandstone ore	Um Bogma formation, Egypt	0.022% U	Organic acids	Percolation column test, 1-kg ore packed in a truncated cone (11 cm × 7 cm top), particle size ≤ 2 mm, 20 days	5.7% U	Abdulla et al. (2018)
*Pseudomonas fluorescens* SHA 281	Sandstone ore (T-2)80	Gabal Gattar, Egypt	0.03% U	Gluconic acid (8.4 g L^−1^)	Shake flasks, 10% PD, 35 °C, 175 rpm, 8 days, two-step process	58% U	Hussien et al. (2016)
*Pseudomonas fluorescens* SHA 281	Waste rock (W-1)	Gabal Gattar, Egypt	0.017% U	Gluconic acid (8.4 g L^−1^)	Shake flasks, 10% PD, 35 °C, 175 rpm, 8 days, two-step process	60% U	Hussien et al. (2016)

^a^PD, ore pulp density. In the one-step leaching process, the bacterial culture and uranium ore are added to the same leaching system and incubated together. In the two-step leaching process, the bacterial culture is pre-cultivated before the ore is added to the medium and then incubated together.

## 4 Bacterial sequestration of uranium

### 4.1 Uranium sequestration with siderophores

Siderophores are low-molecular-weight, high-affinity metal-chelating biomolecules in microorganisms and plants (Hider and Kong, 2010; Anderson et al., 2011; Ahmed and Holmström, 2014; Saha et al., 2016; Bahaloo-Horeh and Sadri, 2025; Schalk, 2025). Siderophores in bacteria are usually hydroxamate-, catecholate-, carboxylate-, and mixed-type molecules with multiple and varied chelates (Gledhill et al., 2004; Mawji et al., 2011; Timofeeva et al., 2022). They contain anionic hydroxamate or catecholate functional groups that form oxo-donors, which bind to hard Lewis acids and form complexes with high stability constants (Ahmed and Holmström, 2014; Padial et al., 2019; Liu et al., 2023; Chukwuma et al., 2025). Siderophores are linked to outer membrane proteins, which transport iron into the cell. They contain several functional chelating groups that form soluble uranyl and other actinide complexes with oxygen (Ivanov et al., 2019; Hofmann et al., 2020; Gomes et al., 2024).

Some siderophores have chelation moieties, including hydroxamic acid [R–C(=O)–N(–OH)–R′], catechol [C_6_H_4_(OH)_2_], and carboxyl (–COOH) groups in the same molecule (Ali and Vidhale, 2013; De Serrano et al., 2016; Xie et al., 2024). Hydroxamate siderophores have the general formula C(=O)N-(OH)R, where “R” represents an amino acid or its derivative, containing two O atoms that form a bi-dentate ligand with Fe^3+^ ions. When hydroxamate combines with Fe^3+^ ions, its functional group loses a proton from the hydroxylamine group (–NOH) to form a bidentate ligand (Butler and Theisen, 2010). The siderophore desferrioxamine B has been shown to form soluble complexes with Pu(IV) from plutonium oxide (PuO_2_) and U(IV) from uraninite (UO_2_) (Frazier et al., 2005; Boukhalfa et al., 2007; Mullen et al., 2007; Février et al., 2021). Uranium complexes with biogenic siderophores are formed over a wide pH range, potentially increasing the mobility of uranium species in environmental settings such as weathering of rocks and groundwater aquifers (Kraemer et al., 2015).

Kalinowski et al. (2004, 2006) investigated the solubilization of uranium from uranium-rich shale samples (kolm in Ranstad, Sweden). The unprocessed kolm sample contained 0.61% U and the acid pre-leached kolm sample contained 0.0013% U. The bioleaching was tested with *P. fluorescens, Pseudomonas stutzeri*, and *Shewanella putrefaciens* cultures at 0.75 and 3% pulp densities for 5 and 8 days of incubation at 21 °C. *S. putrefaciens* dissolved uranium negligibly (0.11% U), and siderophore production was not detected. *P. fluorescens* solubilized 8.1 mg L^−1^ U within 5 days of contact time, equal to 18% U leaching efficiency. *P. fluorescens* also dissolved 0.016%−0.9% U from samples of weathered uranium ore (0.0029% U), acid-preleached ore (0.0099% U), and kolm (0.52% U) in chemically defined media. The dissolution was attributed to siderophore (pyoverdine) sequestration by *P. fluorescens* rather than acid attack because the dissolution increased with pH.

Munir et al. (2019) tested *Myroides odoratimimus* (isolated from a uranium mine), and *Pseudomonas aeruginosa* and *P. fluorescens* (from two Chinese culture collections) in Na-lactate medium in shake flask (120 rpm) leaching experiments with a sample of a hard rock-type uranium ore [0.2496% U, Ø ≤ 73 μm; 0.1965% U(IV) and 0.0531% U(VI)]. The sample was collected from the 721 Uranium Mine (Jiangxi Province, SE-China), which contained pitchblende [(U^4+^U^6+^)O_2_] as the main uranium host mineral. The leach solution contained 20, 12, and 10 mg U L^−1^ after 7 days of incubation at 30 °C at 5% pulp density in cultures of *M. odoratimimus, P. aeruginosa*, and *P. fluorescens*, respectively. The highest leaching efficiency (*M. odoratimimus*) was 15.9% uranium dissolution. Uranium dissolution was mainly attributed to siderophores produced by the bacteria. Qualitative testing indicated that *M. odoratimimus* produced more siderophores in CAS blue agar plates compared with the two *Pseudomonas* species (Munir et al., 2019). CAS blue agar is a diagnostic test for siderophore detection (Louden et al., 2011).

Moll et al. (2008) identified two different uranium–pyoverdin complexes formed by *P. fluorescens*. In the case of pre-leached uranium kolm sample, *P. fluorescens* dissolved 14.3 μg U L^−1^ after 8 days of contact time, representing a leaching efficiency of 3.7% U. *P. stutzeri* and *S. putrefaciens* dissolved 0.36 and 0.31% U, respectively, after 8 days of contact time. Neither strain produced siderophores during the incubation. Uranium solubilization in the respective chemical control was 0.31% U leaching efficiency. The studies suggested that uranium dissolves with time from the shale mineralization, but how the dissolution impacts the locale and environmental quality is not understood in full scope.

Lu et al. (2024) characterized the surface binding of ${\text{UO}}_{2}^{2+}$ in *S. putrefaciens* cells, which precipitated the complex as chernikovite [(H_3_O)_2_(UO_2_)_2_(PO_4_)_2_·6H_2_O]. This complexation was alleviated by siderophore desferrioxamine (DFO) B, because it preferentially chelated the uranyl ion as a soluble U(VI)–DFO complex instead of U(VI) biomineralization with phosphate functional groups. In the presence of Fe^3+^, *S. putrefaciens* precipitated uranium as bassetite [Fe^2+^(UO_2_)_2_(PO_4_)_2_·10H_2_O] as a result of the hexadentate coordination of DFO B with Fe^3+^ and ${\text{UO}}_{2}^{2+}$ via its three hydroxamic functional groups. Yang et al. (2026) noted that *S. putrefaciens* had multiple pathways of uranium sequestration: biosorption by surface functional groups, biomineralization of uranium phosphate complexes, and extracellular and intracellular bioaccumulation in the biomass. *S. putrefaciens* has outer membrane cytochromes, including the Mtr pathway, which can reversibly shuttle intracellular electrons to extracellular electron acceptors in the reductive pathway of cell surface-bound U(VI), as demonstrated by cyclic voltammetry (Yang et al., 2024). Similarly, *Methylobacterium* biofilms removed uranium from the solution phase not only by sorption in the biofilm matrix but also by phosphatase-mediated biomineralization and intracellular accumulation in biofilm cells (Chowdhury et al., 2023). Uranium minerals as biomineralization products and other solid phases that have been characterized in heterotrophic bacterial cultures are listed in Table 1.

Hussien et al. (2013) reported that *P. fluorescens* produced pyoverdine-type siderophores that could sequester 90% U from monazite [(Ce,La,Nd,Th)PO_4_] and 86% U from a thorium–uranium concentrate under optimum conditions. Similarly, Desouky et al. (2016) reported that siderophores purified from *P. aeruginosa* sequestered 68% U and 65% Th in 24 h from a sample of Th–U monazite concentrate (2.44% U, 19.01% ThO_2_, and 20.07% RE_2_O_3_) at 1.2% pulp density, pH 5.3, at 22 °C. The function of pyoverdines in *Pseudomonas* spp. is iron sequestration. Their chemical structure contains one catechol and two hydroxamate ligands that sequester Fe^3+^ (Cézard et al., 2015).

### 4.2 Uranium sequestration with EPS and LPS

Kazy et al. (2008) characterized EPS produced by *P. aeruginosa* (strain BU2) for its capacity to sequester uranium from aqueous solutions. The EPS fraction was acidic, and uranium (as ${\text{UO}}_{2}^{2+}$) biosorption was pH-dependent and ranged from 58 mg U g^−1^ EPS at pH 1.0 to 198–985 mg U g^−1^ EPS at pH 5.0 within 1 h of equilibration. Higher pH values could not be tested because of uranium precipitation. Fourier transform infrared (FTIR) spectroscopy data confirmed the binding of uranium with carboxyl groups of uronic acids or alduronic acids (i.e., glucuronic, galacturonic, mannuronic, and guluronic acids) of bacterial EPS at pH 5. Uranium bound in *Pseudomonas* MTCC 3087 biomass (pH 5.0, 12 h) was sequestered in the cell cytoplasm as electron-dense precipitates, as evident in transmission electron microscopy (TEM) micrographs (Kazy et al., 2009). The binding of uranium was coupled with the displacement of cellular potassium and calcium and involved cellular phosphate, carboxyl, and amino groups. Uranium formed phosphate complexes with cellular phosphate groups, identified by X-ray diffraction (XRD) analysis as uranium(IV) phosphate or uranium(IV) orthophosphate [U_3_(PO_4_)_4_] and uranium mineral phases such as vanmeersscheite [U^6+^(UO_2_)_3_(PO_4_)_2_(OH)_6_·4H_2_O] and metavanmeersscheite [U^6+^(UO_2_)_3_(PO_4_)_2_(OH)_6_·2H_2_O]. However, in the absence of elemental analysis and with no other information available, these specific formulae of phosphate complexes cannot be authenticated at this time.

Choudhary and Sar (2011a,b) reported the maximum loading capacity of *P. aeruginosa* J007 biomass sorption of 275 mg U g^−1^ dry wt at pH 4.0 and with a 6 h contact time. FTIR analysis indicated a synergistic involvement of deprotonated $PO_{4}^{3-}$ and –COOH functional groups in the precipitation of uranium. XRD analysis identified crystallized uranyl metaphosphate [UO_2_(PO_3_)_2_], uranyl phosphate hydrate [(UO_2_)_3_(PO_4_)_2_·H_2_O], and uranium oxide phosphate [U_2_O(PO_4_)_2_] in the cells. TEM micrographs confirmed uranium sequestration within the cell envelope region (Choudhary and Sar, 2011a). Gerber et al. (2016) used *Acidivorax facilis* (formerly *Pseudomonas facilis*) as biosorbent for 0.05–0.1 mM uranyl nitrate solution (12–24 mg U L^−1^), pH 5.0, for up to 48 h contact. Uranium biosorption was concluded to involve passive sequestration in the outer membrane of the cell wall in parallel with active uptake, which extended into the cytoplasm, totaling 130 mg U g^−1^ dry wt.

A *P. fluorescens* strain, isolated from a granitic rock aquifer at the Aspö Hard Rock Laboratory (Oskarshamn, Sweden), sorbed 124 mg U g^−1^ dry wt at an initial uranium concentration of 24 mg U L^−1^, pH 6.0 (Lütke et al., 2012). The time-resolved laser-induced fluorescence spectroscopy (TRLFS) analysis demonstrated that U(VI) was mostly bound to protonated phosphoryl and carboxylic sites of the cell surface. *Pseudomonas* cells immobilized in a gel matrix sequestered 202 mg U g^−1^ dry wt and, under shaking conditions, 252 mg U g^−1^ dry wt, both at pH 5, 24 h contact time, and an initial uranium concentration of 100 mg U L^−1^ (D'Souza et al., 2006).

The main structural EPS components in bacterial biofilms are heterogeneous fractions of proteins, nucleic acids, lipids, and polysaccharides (Sutherland, 2001; Guibaud et al., 2005; Flemming, 2016; Martino, 2018; Zhou et al., 2024). EPS contain functional carboxyl (–COOH), phosphate (–PO_4_), amino (–NH_2_), and hydroxyl (–OH) groups, which act as nucleation sites for dissolved metals (Baker et al., 2010; Zhang J. et al., 2018; Gavrilescu, 2022). Hydrolysis and acid phosphatase–mediated reactions of exocellular DNA in EPS release phosphate, which sequesters uranium and forms crystallized minerals (Hufton et al., 2016). Some outer membrane cytochromes (e.g., MtrC and OmcA in *Shewanella oneidensis* MR-1), which mediate enzymatic uranium reduction, are also localized in the EPS fraction, where the uranyl ion (${\text{UO}}_{2}^{2+}$) is reduced to insoluble U(IV)O_2_ (Marshall et al., 2006). Other related bacterial groups form reduced UO_2_ on the cell surface, with surface-bound cytochromes mediating electron flow. In contrast, Li et al. (2018) reported U(VI) reduction to U(IV)O_2_, coupled with H_2_ oxidation, in the loosely associated EPS fraction of a *Klebsiella* isolate. The role of external and cell surface-associated cytochromes was ruled out, and the reduction represents, therefore, catalysis of an uncharacterized uranium-reductive pathway.

### 4.3 Bacterial reduction of uranium

Uranium reduction by anaerobes produces crystallized UO_2_, which is resistant to re-oxidation in sediment or aquifer environments. However, biological reduction also produces a non-crystallized uranium phase, which is not as stable as UO_2_ and is coordinated to multiple monomeric phosphates or polymeric networks (Alessi et al., 2014). Sulfate-reducing bacteria co-precipitate uranium phosphate phases as well as FeS and other metal sulfides, which may not only adsorb uranium species but also serve as electron donors for U(VI) reduction to U(IV) species (Chen H. et al., 2026). *Anaeromyxobacter dehalogenans* 2CP-C reduced U(VI) to U(IV) with H_2_ as the electron donor (Wu et al., 2006). With nitrate addition, the culture re-oxidized U(IV) to U(VI), coupled with nitrate reduction. U(VI) reduction was inhibited in the cultures amended with citrate or Fe(III)-citrate, suggesting that the sequestered uranyl–citrate complex was stable under these conditions. Zhu et al. (2022) demonstrated in Fe^3+^-containing solutions in soil column enrichment experiments that uranyl ion was biologically reduced upon addition of an organic amendment (in this case, acetate) and formed a solid-phase U(IV)–O–Fe(II), which was oxidized to a U(VI)–PO_4_ precipitate. Mineralogical identification of the precipitates was not undertaken. Analysis of bacterial enrichments suggested the dominance of *Acinetobacter* and *Stenotrophomonas* spp. in the reduced phase and *Aminobacter* spp. and *Caulobacteraceae* in the formation of the U(VI)–phosphate complex.

Ghasemi et al. (2025) and Rastkhah et al. (2026) described a denitrifying *Shewanella azerbaijanica* strain that reduced U(VI) to U(IV) under anaerobic conditions. The source of electrons for uranium (and nitrate) reduction was the anaerobic oxidation of glucose (1 g L^−1^). Nitrate inhibited uranium reduction, but the inhibition could be alleviated by disrupting the *nap* operon (nitrate reductase) with *mtrC* (which encodes part of an outer membrane cytochrome *c*). Almeida et al. (2025) characterized periplasmic cytochromes that mediate the electron flow to U(VI) reduction in *Geotales uraniireducens* (formerly *Geobacter uraniireducens*). These cytochromes have three heme-binding motifs and undergo three consecutive reversible one-electron steps. If the uranyl ion crosses the outer membrane, the reduction by the triheme cytochromes to insoluble UO_2_ prevents uranium from traversing into the cell interior.

Resting cell suspensions of *Shewanella oneidensis* MR-1 and *Bacillus subtilis* were contacted with uranyl nitrate, which precipitated as hydrogen uranyl phosphate tetrahydrate (HUO_2_PO_4_·4H_2_O), belonging to the autunite/chernikovite group, throughout the cell wall (Dunham-Cheatham et al., 2011). The abiotic precipitates in the absence of bacteria were also comparable, but they were more abundant and of a larger size. The smaller particles in bacterial cells had larger surface areas and therefore a somewhat increased solubility. Additionally, lysis in resting cell suspensions released excreta that sequestered uranium in the solution phase; therefore, the abiotic precipitates were more abundant under otherwise similar conditions (Dunham-Cheatham et al., 2011).

Some *Shewanella* and *Geobacter* cells have pili that have electron-conducting properties (Orellana et al., 2013). Pili in *Geobacter sulfurreducens* conduct electrons from periplasmic cytochromes to uranyl ions, immobilizing U(IV) on the cell surface (Clark et al., 2021). Additionally, in *G. sulfurreducens*, uranium is also sequestered within the LPS layer on the cell surface, thus protecting the outer membrane from contact with uranium (Clark et al., 2021). External electron transfer from the cell to U(VI) in *Desulfovibrio vulgaris* UR1 is facilitated by magnetite (Fe^2+^${\text{Fe}}_{2}^{3+}$O_4_) and biochar because they can function as electron shuttles along with extracellular oxidoreductases and Fe–S proteins involved in charge transfer (Xu et al., 2025). U(VI) reduction by *D. vulgaris* UR1 in a simulated groundwater aquifer maintained >90% reduction of 50 μmol U L^−1^ (11.9 mg U L^−1^) solution flow over a 30-day time course (Jiang et al., 2026). The relative extent of uranium reduction could be controlled with the flow rate. The reductive activity was effectuated by external electron transport associated with EPS of *D. vulgaris* UR1. The bacteria were in biofilm aggregates on inert quartz sand, and the reduced uranium was in nanoparticle-sized deposits on quartz sand surfaces as well as in the outflow. Jiang et al. (2026) noted that, apart from UO_2_, no other biomineralized U(IV) phase was detected with XRD analysis. In a field experiment with injection wells for adding *D. vulgaris* UR1 and monitoring wells located 10 m downstream, the extent of U(VI) removal was sustained at < 10% residual of the initial 10 μmol U L^−1^ (2.4 mg U L^−1^) in the groundwater even for periods without an external carbon source (Jiang et al., 2026). In the absence of a mass balance analysis, the role of uranium bioreduction remains unclear in this field-scale trial.

Zhou et al. (2014) suggested uranium reduction also by bacterial spores in a *Clostridiales*-dominated fermentative membrane biofilm culture. After contact with U(VI), spores were coated with a thin layer of uranium precipitates. Mature bacterial spores have a proteinaceous shell, the exosporium, which varies in the number of different proteins in spore-formers. Although Zhou et al. (2014) did not further elucidate the role of spores in uranium reduction, other studies have provided evidence for the role of spore proteins in sustained U(VI) reduction (Vecchia et al., 2010; Ontiveros-Valencia et al., 2017; Hao et al., 2025). These findings may be of relevance, for example, in uranium-contaminated oligotrophic groundwater aquifers and other extreme environments where spore-formers may not have sufficient nutrient levels or other growth conditions for germination but can still be active in uranium biotransformations. As shown with *Bacillus subtilis* strain 168, spore adsorption of uranium (added as uranyl nitrate) was ascribed to the carboxyl and amino functional groups on the spore surface, while phosphate sequestration was of lesser significance (Hao et al., 2025).

Indryati et al. (2025) isolated several *Bacillus* cultures from samples of radioactive britholite [(Ce,Ca,Th,La,Nd)_5_(SiO_4_,PO_4_)_3_(OH,F)] mineralization collected in Mamuju, Sulawesi Selatan, Indonesia. The sample of naturally occurring radioactive materials contained 3.582% Th and 1.057% U. The radionuclide activity in the sample was 205,049 Bq kg^−1^ ^232^Th and 32,450 Bq kg^−1^ ^238^U. One isolate, strain L0A, was resistant to at least 1,500 mg U L^−1^. Tentative bioleaching tests with the isolate at an alkaline pH range of 8.0–8.3 yielded dissolution of 2.5 mg U L^−1^ from a uraniferous britholite sample after 5 days of contact time (Indryati et al., 2025). Uranium dissolution at 2.5 mg L^−1^ appears to bear no relevance to the resistance to the 600-fold higher uranium concentration in the culture solution. Although uranium resistance has been screened in many bacteria (Bencheikh-Latmani and Leckie, 2003; Gao et al., 2019), its specific biochemical and molecular biological basis in prokaryotes is not fully resolved. The affinity of uranium to cellular fractions and its many effects on growth, the electron transport chain, and energy transduction require multiple sequestration and biochemical mechanisms such as antioxidants, stress proteins, substrate binding proteins, permeases, transport regulators, and efflux pumps to protect cell membranes and cytoplasm (Yung et al., 2015; Rogiers et al., 2022; Pathak et al., 2017). Gallois et al. (2022) identified in uranium-resistant soil bacteria a cell surface protein UPiA that had a high affinity for uranyl ion as well as Fe^3+^. It was tentatively concluded that the UPiA was expressed when the cells were exposed to uranium, and binding of uranium with UPiA effectively prevented its transit into the cell interior.

Uranyl ion formed surface complexes with neutral phosphate and deprotonated carboxyl functional groups of *B. subtilis* cell wall at pH 5.0 (Fowle et al., 2000). In *P. fluorescens*, the cell wall served as a ligand for uranyl species [${\text{UO}}_{2}^{2+}$, UO_2_OH^+^, (UO_2_)_2_${\text{(OH)}}_{2}^{2+}$, and UO_2_Cl^+^] due to the negative cell surface charge. TEM micrographs showed that uranyl was associated with the outer and cytoplasmic membranes and deposited along the periplasm separating the two membranes (Bencheikh-Latmani and Leckie, 2003). The metabolic activity, measured as glucose and citrate oxidation by uranium-loaded cells, was greatly suppressed, but removal of uranium with bicarbonate (a strong ligand for uranyl ion) removed most cell-bound uranium and partially relieved metabolic inhibition. Biomineralization with phosphates, sequestration with other functional groups, and reduction of U(VI) all change uranium bioavailability and thereby partially alleviate the toxicity of uranyl ion to metabolically active cells (Rogiers et al., 2022).

## 5 Biomass sorption, bioprecipitation, and biomineralization of uranium

### 5.1 *Bacillus* spp.

The sorption capacity of uranium in *Bacillus* biomass is in the range of 6.3–377 mg U g^−1^ biomass (Hoyle-Gardner et al., 2023). The sequestration involves monolayer adsorption and functional phosphate, hydroxyl, amino, and carboxyl groups on the cell wall. The pH and the presence of other metals in the solution phase influence the sequestration. Qiang et al. (2025) used a deep learning neural network and sensitivity analyses to identify the five most important factors affecting uranium sequestration in bacterial biomass: contact time, temperature, initial uranium concentration, pH, and biomass concentration.

*Bacillus thuringiensis* isolates from U-contaminated soil samples were shown to accumulate uranium, added as uranyl acetate, up to 400 mg U g^−1^ biomass dry wt (Pan et al., 2015). Uranium was initially adsorbed on the cell surface, involving sequestration with phosphate, amino, and methylene functional groups, followed by transformation to uramphite [(NH_4_)(UO_2_)PO_4_·3H_2_O]. Uranium sequestration by coordination with functional groups on the cell surface was also consistent with other *Bacillus* soil isolates (Zhong et al., 2021). The sequestration was non-reductive and, over time, formed other solid phases such as chernikovite [(H_3_O)_2_(UO_2_)_2_(PO_4_)_2_·6H_2_O], autunite [Ca(UO_2_)_2_(PO_4_)_2_·10-12H_2_O], saléeite [Mg(UO_2_)_2_(PO_4_)_2_·10H_2_O], and ankoleite [K_2_(UO_2_)_2_(PO_4_)_2_·6H_2_O], depending on the experimental conditions (Song et al., 2019). These solid uranium phases are listed in Tables 1 and 3. Additional examples of uranium biomineralization have been presented by Chen Y. et al. (2026).

**Table 3:** Summary of uranium mineral and other solid-phase formation during the bioprecipitation/biomineralization of uranyl ion by heterotrophic bacteria.

Mineral	Formula	Bacteria	Reference
Ankoleite	K_2_(UO_2_)_2_(PO_4_)_2_·6H_2_O	*Bacillus* isolate	Song et al. (2019)
Autunite	Ca(UO_2_)_2_(PO_4_)_2_·10-12H_2_O	*Bacillus* isolate	Song et al. (2019)
		*Rahnella* isolate	Beazley et al. (2007)
		*Pseudomonas fluorescens*	Bencheikh-Latmani et al. (2003)
		*Microbacterium oxydans*	Merroun et al. (2006)
		*Sphingomonas* sp.	Merroun et al. (2006)
Bassetite	Fe^2+^(UO_2_)_2_(PO_4_)_2_·10H_2_O	*Shewanella putrefaciens*	Lu et al. (2024)
Chernikovite	(H_3_O)_2_(UO_2_)_2_(PO_4_)_2_·6H_2_O	*Acinetobacter* YU-SS-SB-29	Sowmya et al. (2014)
		*Bacillus* isolate	Song et al. (2019)
		*Enterobacter* N1-10	Yu et al. (2024)
		*Shewanella putrefaciens*	Lu et al. (2024)
Hydroxyapatite	Ca_5_(PO_4_)_3_OH	*Enterobacter* N1-10	Yu et al. (2024)
Meta-autunite	Ca(UO_2_)_2_(PO_4_)_2_·6H_2_O	*Idiomarina loihiensis* MAH1	Morcillo et al. (2014)
		*Caulobacter crescentus*	Yung and Jiao (2014)
		*Chryseobacterium* sp. (PMSZPI)	Khare and Acharya (2024)
		*Rhodanobacter* A2-61	Sousa et al. (2013)
		*Bacillus sphaericus*	Merroun et al. (2011)
		*Serratia* sp. (OT II 7)	Chandwadkar et al. (2018)
		*Sphingomonas* sp.	Merroun et al. (2011)
Metanatroautunite	NaUO_2_PO_4_·3H_2_O	*Brevundimonas vesicularis* LWG1	Liang et al. (2025)
Metavanmeersscheite	U^6+^(UO_2_)_3_(PO_4_)_2_(OH)_6_·2H_2_O	*Pseudomonas* sp. (MTTC 3087)	Kazy et al. (2009)
Ningyoite	(U,Ca,Ce)_2_(PO_4_)_2_·1-2H_2_O	Natural bacterial community	Wang et al. (2023)
Saléeite	Mg(UO_2_)_2_(PO_4_)_2_·10H_2_O	*Bacillus* isolate	Song et al. (2019)
		Natural bacterial community	Wang et al. (2023)
Uranyl phosphate tetrahydrate	(UO_2_)_3_(PO_4_)_2_·4H_2_O	*Bacillus* sp. (dw-2)	Tu et al. (2019)
Uramphite	(NH_4_)_2_(UO_2_)_2_(PO_4_)_2_·6H_2_O	*Bacillus* isolate	Zhong et al. (2021)
		*Bacillus cereus* 12-2	Zhang J. et al. (2018)
		*Bacillus thuringiensis* X-27	Zhu et al. (2023)
Uraninite	UO_2_	*Serratia* sp.	Newsome et al. (2015)
Uranyl metaphosphate	UO_2_(PO_3_)_2_	*Pseudomonas aeruginosa* J007	Choudhary and Sar (2011a)
Uranyl phosphate hydrate	(UO_2_)_3_(PO_4_)_2_·H_2_O	*P. aeruginosa* J007	Choudhary and Sar (2011a)
Uranyl hydrogen phosphate	UO_2_HPO_4_·4H_2_O	*Bacillus* sp. dw-2	Tu et al. (2019)
		*Bacillus subtilis*	Dunham-Cheatham et al. (2011)
		*Serratia sp*. (OT II 7)	Chandwadkar et al. (2018)
		*Shewanella oneidensis* MR-1	Dunham-Cheatham et al. (2011)
Uranium oxide phosphate	U_2_O(PO_4_)_2_	*P. aeruginosa* J007	Choudhary and Sar (2011a)
Uranium(IV) phosphate	U_3_(PO_4_)_4_	*Pseudomonas* sp. (MTTC 3087)	Kazy et al. (2009)
Uvanite	U_2_V_6_O_21_·15H_2_O	*Burkholderia fungorum*	Koribanics et al. (2015)
Vanmeersscheite	U^6+^(UO_2_)_3_(PO_4_)_2_(OH)_6_·4H_2_O	*Pseudomonas* sp. (MTTC 3087)	Kazy et al. (2009)

The references for these data have not reported elemental analyses for these mineral phases. All mineral data have been normalized to formulae at https://www.mindat.org.

*Bacillus sphaericus* JG-A12 was isolated from a uranium mining waste pile near Johanngeorgenstadt in Saxony, Germany (Merroun et al., 2005). The cells were enveloped by a highly ordered crystallized proteinaceous surface layer (S-layer), which could bind uranium and other metals. The X-ray absorption fine-structure (XAFS) analysis showed that ${\text{UO}}_{2}^{2+}$ is coordinated to carboxyl groups (–COOH) in a bidentate fashion, with an average distance between the U atom and the C atom of 2.88 ± 0.02 Å, and to phosphate groups (${\text{PO}}_{4}^{3-}$) in a monodentate fashion, with an average distance between the U atom and the P atom of 3.62 ± 0.02 Å. TEM micrographs showed that uranium accumulated by the *B. sphaericus* strain was located in dense deposits on the cell surface (Merroun et al., 2005).

Panak et al. (2000) tested several *Bacillus* isolates from tailings of a uranium mine for uranium sequestration in the range of 11–214 mg U L^−1^. Uranium sequestration increased with uranium concentration in the solution phase and reached about 200 mg U g^−1^ dry wt biomass. Some *Bacillus* spores reached 20%−25% higher uranium biomass sorption than vegetative cells. Uranium formed uranyl–phosphate complexes with cell wall constituents and could be eluted with EDTA–Tris extraction (Panak et al., 2000). The removal efficiency varied with the *Bacillus* strain. The highest (85%) uranium accumulation was observed with *B*. *megaterium* strain B5385 (Panak et al., 2000). Gorman-Lewis et al. (2005) characterized uranyl surface complexes with hydroxide, calcium carbonate, and carbonate functional groups on *B. subtilis*. The binding of the uranyl ion varied with the binding site and pH. The pH range of 7–9 yielded the highest extent of adsorption. Alkaline-earth–uranyl–carbonate complexes are considered particularly relevant in uranium-contaminated groundwater aquifers (Kerisit and Liu, 2010).

Several other studies have screened *Bacillus* cultures for uranium sequestration (Tsuruta, 2006; Choudhary and Sar, 2011b; Li et al., 2014; Chowdhury et al., 2024). A *Bacillus* isolate was shown to dissolve phosphate from tricalcium phosphate [Ca_3_(PO_4_)_2_] and β-glycerophosphate, resulting in 38%−67% uranium sequestration as poorly soluble phosphate complexes (Chowdhury et al., 2024). Parallel biomass sorption (100 mg U L^−1^, pH 4.0, 14 days) was 3 mg U g^−1^ biomass, suggesting preferential equilibration with phosphate groups. Another *Bacillus* isolate sequestered 24 mg U g^−1^ biomass at pH 7.0 and 20 °C over a 3 h contact time, attributed to monolayer adsorption as well as –OH, –NH_2_, and –COOH functional groups (Hoyle-Gardner et al., 2023). *Bacillus velezensis* strain UUS-1 concentrated uranium from seawater and reached 9.46 ± 0.39 mg U g^−1^ dry wt within 48 h (Yuan et al., 2019). *Bacillus amyloliquefaciens* sequestered 179 mg U g^−1^ biomass (initial uranium concentration of 200 mg U L^−1^ uranyl nitrate, pH 6.0, 3 h) with hydroxyl, amino, and carboxyl functional groups on the surface of the cell wall, as confirmed by X-ray photoelectron spectroscopy (XPS) and FTIR (Liu et al., 2019). Additional uranium biomass sorption data are presented in Table 4.

**Table 4:** Summary of the cellular sequestration of uranium by heterotrophic bacteria.

Bacterial strain	U solution (mg U L^−1^)	pH	Contact time (h)	U bound (mg U g^−1^ dry wt)	Reference
*Acidivorax facilis*	11.9–23.8	5.0	8, 24	130	Gerber et al. (2016)
*Arthrobacter nicotianae* IAM12342	125	5.8	1	615	Tsuruta (2002)
*Bacillus* sp.	125	5.8	1	298–467	Tsuruta (2002)
*Bacillus* ZJ-3	50	7.0	1	35	Zhong et al. (2021)
*Bacillus cereus* 12-2	800	5.0	48	449	Zhang J. et al. (2018)
*Bacillus isolate* MRS-1	50	7.0	3	24.5	Hoyle-Gardner et al. (2023)
*Bacillus amyloliquefaciens*	200	6.0	3	179	Liu et al. (2019)
*Bacillus aryabhattai* TPO3	5	9.2	6	4.3	Banala et al. (2021b)
*Bacillus thuringiensis* BRC-ZYR4	100	5.5	12	420	Pan et al. (2015)
*Bacillus velezensis* UUS-1	10	7.0	24	48.25 ± 5.6	(Yuan et al. (2019)
	100	7.0	24	353.4 ± 15.3	
*Chryseobacterium* sp. (PMSZPI)	238	7.0	24	350	Khare and Acharya (2024)
*Magnetospirillum magneticum* AMB-1	24	6.5	3–4	22	Krawczy-Bärsch et al. (2022)
*Pantoea* sp. (TW18)	40	4.1	24	80	Zhang Z. et al. (2018)
*Pseudomonas* sp.	100	5.0	24	202	D'Souza et al. (2006)
*Pseudomonas* sp. (active cells)	100	5.0	24	245 ± 15.5	Sar and D'Souza (2001)
*Pseudomonas* sp. (lyophilized cells)	100	5.0	24	252 ± 7.6	Sar and D'Souza (2001)
*Pseudomonas aeruginosa* J007	100	4.0	6	275	Choudhary and Sar (2011a,b)
*Pseudomonas fluorescens*	24	6.0	48	124	Lütke et al. (2012)
*Rhodanobacter* A2-61	119	5.0	24	29	Sousa et al. (2013)
*Serratia* sp. (OT II 7)	238	5.0	120	232	(Chandwadkar et al. (2018)
	238	7.0	120	360	
	238	9.0	120	376	
*Staphylococcus aureus* LZ-01	476	5.0	6	428	Zou et al. (2014)
*Streptomyces levoris*	11.9	3.5	1	69	Nakajima and Tsuruta (2002)
*Streptomyces sporoverrucosus*	10	3.0	12	15	Li et al. (2016)

*Bacillus aryabhattai* sequestered ~70% of the uranium in 6 h with a loading capacity of 4.3 mg U g^−1^ dry wt in 1 mM carbonate–bicarbonate solution at pH 9.2 (Banala et al., 2021a). This was confirmed with scanning electron microscopy and energy-dispersive X-ray fluorescence spectroscopy of the biomass. FTIR results confirmed the uranium sequestration with phosphate, amino, and carboxyl functional groups in the cell membrane. TEM micrographs showed the presence of uranium precipitates also in the cytoplasmic fraction. *Bacillus cereus* 12-2 immobilized U(VI) in a two-phase time course of relatively rapid sequestration (30 min) of uranium with carboxyl, amino, and phosphoryl groups on the cell surface as amorphous complexes, followed by mineralization of uranium as a crystallized uramphite phase [(NH_4_)_2_(UO_2_)_2_(PO_4_)_2_·6H_2_O] during metabolic activity over 48 h. The biomass reached 449 mg U g^−1^ dry wt at pH 5 from an initial uranium concentration of 800 mg U L^−1^ (Zhang Z. et al., 2018). Tsuruta (2002) tested uranium sorption in several *Bacillus* species, and it ranged from 297 to 467 mg U g^−1^ dry wt biomass. *B. thuringiensis* isolates from U-contaminated soil samples were shown to accumulate uranium, added as uranyl acetate, up to 400 mg U g^−1^ dry wt (Pan et al., 2015).

Uranium has a high affinity for nucleic acids and other cellular fractions, and its presence in the cytoplasmic (soluble) fraction does not imply active metabolism-dependent bioaccumulation of uranium. The variation in uranium sorption in biomass of *Bacillus* spp. is considerable, even 50–60-fold (Table 4), and it is not clear what specific cellular properties account for this range. It is also possible that laboratory practices of preparation and washing of cell suspensions may remove functional groups from cell envelopes. The chemical composition of the solution phase is also a variable. In this regard, experimental protocols, e.g., contact time, pH, temperature, and uranium concentration, are variables in uranium sequestration. These empirical studies have not been integrated and advanced to thermodynamic analysis and optimization of experimental parameters for possible biotechnology applications.

### 5.2 Other heterotrophic bacteria

Uranium sorption experiments with *Streptomyces levoris* and several other species yielded biomass loading of 17–69 mg U g^−1^ g dry wt. within 1 h contact time from 50 μM U [11.9 mg U L^−1^]. In the solution phase at pH 3.5, 96.9% of U(VI) is the ${\text{UO}}_{2}^{2+}$ species [2.9% UO_2_(OH)^+^, 0.2% (UO_2_)_2_${\text{(OH)}}_{2}^{2+}$] (Nakajima and Tsuruta, 2002). *Streptomyces sporoverrucosus* dwc-3 strain, isolated from a uraniferous radioactive waste disposal site (SW China), reached equilibrium in uranium sorption in 12 h at pH 3.0, with amino, phosphate, and carboxyl groups as ligands on the cell walls and within the cell (Li et al., 2016). Proton-induced X-ray emission (PIXE) and enhanced proton backscattering spectrometry (EPBS) data indicated that uranium sorption also involved an ion-exchange mechanism, whereby potassium is released following uranium sorption. Whether this is a chemical exchange to maintain electroneutrality or a biologically mediated process remains unclear.

The experimental time courses of uranium biosorption by bacterial cells vary greatly. Tsuruta (2002) reported that *Arthrobacter nicotianae* IAM12342 sequestered 615 mg U g^−1^ dry wt biomass from a solution containing 125 mg U L^−1^ at pH 5.8, with 1 h contact time. Zou et al. (2014) showed that *Staphylococcus aureus* strain LZ-01 sequestered over 90% U from a 476 mg U L^−1^ aqueous solution (6 h, 37 °C), and this was clearly enhanced with the addition of 20 mmol L^−1^ phosphate. This LZ-01 strain is active at pH 5.0 (Zhang et al., 2013). *Magnetospirillum magneticum* AMB-1 sequestered over 90% U(VI) from a 24 mg U L^−1^ aqueous solution within 3–4 h. Much of the uranium binding was attributed to peptidoglycan (i.e., mostly carboxyl and amino functional groups) in the outer membrane of the cell wall (Krawczy-Bärsch et al., 2022).

Shukla et al. (2020) concluded that acid phosphatase was key in uranium phosphate precipitation is *S. aureus* biofilms. Bacterial activity and metabolism are key factors in uranium transformation in biofilms. Established biofilms have microenvironments that vary with respect to spatial depth in pH, redox, nutrients, physiological and genetic traits, metals, and dissolved oxygen conditions. Non-invasive analytical techniques are important tools in characterizing these differences (Arnold et al., 2010). Uranium speciation in such differential microzones may involve sequestration with available functional groups, oxidation-reduction reactions, cellular toxicity, and metabolic inhibition.

*Brevundimonas vesicularis* strain LWG1, a U-resistant bacterium isolated from uranium mine wastewater, precipitated uranium in extracellular phosphate complexes (Liang et al., 2025). X-ray diffraction and high-angle annular dark-field scanning TEM techniques confirmed the extracellular precipitation of uranyl phosphate as metanatroautunite (NaUO_2_PO_4_·3H_2_O). Fourier transform infrared spectroscopy (FTIR) revealed that sequestration was facilitated by carboxyl, hydroxyl, amino, and phosphate functional groups on the bacterial cell surface. X-ray photoelectron spectroscopy indicated a partial reduction of U(VI) to U(IV) by *B. vesicularis* LWG1 strain, facilitated by a reductive environment (Liang et al., 2025). *Caulobacter crescentus* precipitated uranyl phosphate on the cell surface in the form of meta-autunite [(Ca(UO_2_)_2_(PO_4_)_2_·6H_2_O)] (Yung and Jiao, 2014). Martínez-Rodríguez et al. (2023) showed that a *Microbacterium* strain precipitated uranium phosphate on the cell surface with an external phosphate source. In the absence of an external source, about 60% of uranium was sorbed into the biomass within a matter of minutes, and with time, uranium formed intracellular phosphate crystals with phosphate available in biomolecules such as proteins and nucleotides in the cytoplasm.

Periplasmic alkaline phosphatase activity (PhoY) binds inorganic phosphate for uranium sequestration and may act as a barrier to prevent the entry of uranium into the cytoplasm. Two outer membrane transporters in *C. crescentus* were identified as having roles in uranium efflux and maintaining membrane integrity (Yung et al., 2015). Uranium toxicity in *C. crescentus* was manifested as a metabolic uncoupling effect and inhibition of replication (Park and Jiao, 2014; Yung et al., 2015). Column (12.5 cm by 5 cm) studies by Beazley et al. (2011) provided evidence that phosphatase activity released inorganic phosphate from glycerol phosphate for the precipitation of a uranium phosphate phase.

A *Serratia* isolate (strain OT II 7) derived from the uranium mineralization at Domiasiat, NE-India, precipitated uranyl phosphate from uranyl nitrate (pH 5) and uranyl carbonate (pH 7 and 9) by hydrolyzing Na-β-glycerophosphate with acid and alkaline phosphatases (Chandwadkar et al., 2018). The precipitates formed on the cell wall and comprised meta-autunite [Ca(UO_2_)_2_(PO_4_)_2_·6H_2_O] and uranyl hydrogen phosphate [UO_2_HPO_4_·4H_2_O]. Meta-autunite was not formed at pH 7 and 9. *Rhodanobacter* A2-61, isolated from the Urgeiriça Mine, Portugal, sequestered about 120 μmol U L^−1^ (29 mg U L^−1^) within 24 h when grown aerobically in the presence of 500 μmol L^−1^ U (119 mg U L^−1^) at pH 5 (Sousa et al., 2013). Scanning electron microscopy–energy-dispersive spectroscopy (SEM–EDS) analysis of heated cells showed the presence of phosphate and uranium in the mass, suggesting their precipitation within the cells. XRD analysis of uranium-loaded cells identified meta-autunite, uranyl phosphate [UO_2_(PO_4_)_2_], and uranyl phosphate hydrate [(UO_2_)_3_(PO_4_)_2_·H_2_O] as solid phases.

Meta-autunite formation was also reported by Pinel-Cabello et al. (2025) in *Stenotrophomonas bentonitica* cultures. Cells formed uranium phosphates on cell wall as acid and alkaline phosphatase activity hydrolyzed organophosphates, concurrent with the formation of meta-autunite crystals on the cell surface. The precipitation prevented uranium from reaching the cell membranes and cytoplasm. Pinel-Cabello et al. (2021, 2025) concluded that the very first step was passive sorption of uranium on nucleation sites of organophosphate, before phosphatase activities released phosphate for precipitation as meta-autunite. Associated with exposure to uranium was upregulation of multiple genes for phosphatases, cell wall synthesis, and select transport proteins.

*Enterobacter* N1-10 produced carboxylic acids (lactic, citric, succinic, and D-glucuronic acids), which solubilized Ca_3_(PO_4_)_2_ and precipitated uranyl ion as chernikovite and hydroxyapatite [Ca_5_(PO_4_)_3_OH] phases (Yu et al., 2024). Hydroxyapatite can also serve as a phosphate source for uranium precipitation (Tan et al., 2025). He et al. (2022) reported that *Pseudomonas* isolates produced 3–10 mg L^−1^ lactic acid, sufficient to dissolve phosphate from Ca_3_(PO_4_)_2_, which then precipitated uranium (15–20 mg U L^−1^) as chernikovite. *Acinetobacter* YU-SS-SB-29, isolated from a monazite sand deposit, solubilized 952 mg $PO_{4}^{3-}$ L^−1^ from Ca_3_(PO_4_)_2_ in Pikovskaya's medium, which contains dextrose and yeast extract as the main carbon sources (Sowmya et al., 2014). Uranium precipitation was tested at different concentrations of ${\text{UO}}_{2}^{2+}$ in spent culture supernatant that initially contained phosphate solubilized from tricalcium phosphate. The precipitate was identified as chernikovite (Sowmya et al., 2014). The precipitation involved initial phosphate dissolution by organic acids produced by *Acinetobacter*. The availability of phosphate in the solution phase is extremely low in the environment because it usually precipitates as organic or inorganic phosphates and polyphosphate complexes (Shen et al., 2011; Kulkarni et al., 2016).

Uranyl ion precipitation with phosphate by *Bacillus* dw-2 was reported to form uranyl hydrogen phosphate (UO_2_HPO_4_·4H_2_O) and uranyl phosphate tetrahydrate [(UO_2_)_3_(PO_4_)_2_·4H_2_O] crystallized solid phases (Tu et al., 2019). The presence of organic acids, especially oxalate and citrate, inhibited uranyl phosphate complexation. Inorganic phosphate was released by anaerobic hydrolysis of intracellular polyphosphates in *Cellulomonas* strain ES6. Inorganic phosphate acted as a ligand for the formation of insoluble uranyl phosphate precipitates during aerobic growth (Sivaswamy et al., 2010). X-ray absorption fine-structure analysis showed that the uranium precipitates consisted primarily of U(VI)–phosphate complexes. High-resolution TEM and energy-dispersive X-ray spectroscopy showed extracellular and intracellular accumulation of solids containing uranium and phosphate with nanometer-sized structures. The reduction of U(VI) to U(IV) was also observed.

Merroun et al. (2011) characterized uranium precipitation by *Bacillus sphaericus* and a *Sphingomonas* sp. at low pH values. X-ray absorption spectroscopy results showed that uranium precipitated both outside and inside the cells as a uranyl phosphate phase of the meta-autunite group. Beazley et al. (2007) showed that a *Rahnella* isolate hydrolyzed organophosphate (glycerol-3-phosphate) and precipitated 73%−95% U as uranyl phosphate over 5 days of incubation in a simulated groundwater experiment. The solid phase was identified as autunite/meta-autunite. The bacteria yielded the highest rates of uranium precipitation and phosphatase activity in the pH range of 5–7. *Streptomyces* spp. have been isolated from abandoned uranium tailings that can hydrolyze inorganic phosphate from lecithin (a varied mixture of phospholipids), leading to uranium phosphate precipitation also inside the cells (Hu et al., 2024). Robinson et al. (2025) demonstrated in microcosm experiments with aquifer sediment samples spiked with uranyl chloride that Ca-citrate + Na-phosphate amendment immobilized uranium in poorly soluble uranyl phosphate precipitates, and it was also sequestered in Ca-phosphate phases. Glycerol phosphate without Ca-citrate amendment was less effective in immobilizing uranium because only uranyl phosphate was formed without sequestration in the Ca-phosphate phase.

Reductive removal of uranium as U(IV) by precipitation was reported by Wang et al. (2023) using a natural bacterial community (mostly *Dysgonomonas, Propionispora, Macellibacteroides*, unclassified *Rhizobiaceae, Propionicimonas*, and unclassified *Rhodocyclacea*). The reduction of U(VI), ${\text{NO}}_{3}^{-}$, and ${\text{SO}}_{4}^{2-}$ by the bacterial community was stimulated by glycerol, and glycerol phosphate enhanced uranium precipitation at pH 5.0. The XRD, SEM–EDS, and XPS results showed the formation of crystallized uranium precipitates as saléeite [Mg(UO_2_)_2_(PO_4_)_2_·10H_2_O] and ningyoite [(U,Ca,Ce)_2_(PO_4_)_2_·1-2H_2_O] mineral phases (Wang et al., 2023).

Yuan et al. (2023) characterized the diversity and richness of heterotrophic bacteria found in pre- and post-bioleaching samples of columns used for acid ferric-iron bioleaching of uranium. The dominant heterotrophic bacteria were distributed among the genera *Pseudomonas, Brevundimonas, Acinetobacter, Bacillus*, and *Clostridium*, and most were associated with the surfaces of ore particles. Their proportions varied, in some cases sharply, depending on pre- or post-leaching sampling. Biomass sequestered uranium from the solution phase, but their active function in the acid ferric sulfate bioleaching process remains unclear. Similarly, heterotrophic bacteria (*Pseudomonas, Brevundimonas, Bradyrhizobium, Rhizobium, Sphingomonas*) were identified in uranium ore samples from the Ranger Mine (Jabiru, NT, Australia) during acid ferric sulfate bioleaching (Vázquez-Campos et al., 2017). The Ranger Mine rehabilitation program is long-standing and has included multiple microbiological surveys. Sutcliffe et al. (2017) incubated surface sediment samples from the Ranger Mine site and spiked them with 0–4,000 mg U kg^−1^ as uranium sulfate, followed by incubation for nearly 20 weeks. At 1,500 mg U kg^−1^ dry sediment, methanogenic consortia were more prevalent than at lower concentrations, based on analysis of gene abundance for methanogenesis and nitrogen fixation. In contrast, heterotrophic metabolism was more sensitive to spiked uranium levels. A spike of 4,000 mg U kg^−1^ dry sediment was overall suppressive. Thus, some components of carbon and nitrogen cycling are not uniformly sensitive to uranium. It is not clear, however, whether such findings could improve management approaches for the rehabilitation of uranium-contaminated mine sites.

In uranium-contaminated natural microbial communities, algal exudates can influence bacterial species composition via nutrient exchange and signaling compounds while also enhancing uranium sequestration (He et al., 2024). In soil systems, uranium sequestration reduces the available phosphate pool, but biodegradation of soil organic matter can partially replenish available phosphate (Yu et al., 2025). In anaerobic reducing zones of alluvial aquifer sediments, nitrate influx can oxidize organic matter–sorbed U(IV), resulting in desorption and release of uranium as mobile U(VI) species (Westrop et al., 2023; Nitzsche et al., 2026). Nitrite, the first detectable intermediate in nitrate reduction and denitrification, is also an abiotic oxidant of U(IV).

Koribanics et al. (2015) isolated a U(VI)-reducing *Burkholderia* sp. from subsurface sediments at the Integrated Field-Scale Subsurface Research Challenge Site in Rifle (CO, United States) (https://lmpublicsearch.lm.doe.gov/lmsites/47149_rfn_vmr-2022-2023.pdf). The site has a long history of uranium contamination due to a now-abandoned uranium–vanadium mill, and various remediation approaches have been tested over the years (Campbell et al., 2011). The isolate, identified as *B. fungorum*, used acetate as the electron donor and U(VI) as the electron acceptor. Biological reduction of U(VI) to U(IV) is an immobilization approach but may have limited use in site remediation because acetate is readily consumed in natural microbial communities. In uranium-contaminated sites, however, as noted by Tang et al. (2021), the biogenic formation of UO_2_ and crystallized uranium mineral phases decreases the bioavailable and inhibitory fraction of uranium and may facilitate the development of diverse microbial communities. Boonchayaanant et al. (2008) used ethanol and acetate to select for soil-borne sulfate reducers. Subsequent kinetic analysis showed that uranium reduction by the enrichment culture was not stoichiometric and indicated co-metabolism, with activity decreasing over time. Biological uranium reduction was insignificant at 10 °C, and the rate constant (L mg cells^−1^ day^−1^) increased 3-fold when the incubation temperature was increased from 20 to 30 °C. The ambient low temperature of topsoil, eluviation layers, and subsoil and groundwater aquifers is a major limiting factor for *in situ* remediation strategies. As the model developed by Zhao et al. (2011) for *in situ* uranium bioreduction demonstrates multiple multi-scale contributing factors, the integration of dynamic biological mechanisms and capacities with reactive transport and hydrological processes is complex and requires extensive simulation of kinetics and quantification of interactive effects at molecular, cellular, and community levels.

Permeable reactive barriers are subsurface trenches for the treatment of groundwater contaminated with toxic metals and plumes of highly chlorinated hydrocarbons (Naftz et al., 2003; Gavrilescu et al., 2009). Trenches are positioned downstream from the source and have zero-valent iron as a reductant with nanoscale particle size and high surface area, typically >20–25 m^2^ g^−1^. Constructed wetland systems with permeable reactive barriers have been used to treat uranium mine water and tailings leachates (Morrison et al., 2006; Lin et al., 2026). Contact with zero-valent iron reduces U(VI) abiotically to U(IV), precipitating as UO_2_. Sulfate reducers and other anaerobes are found in permeable reactive barriers depending on the presence of organic matter (England, 2006).

Laboratory simulations have demonstrated that sulfate reducers enhance the process by direct reduction of U(VI) and by biogenic sulfide acting as a reductant, maintaining low redox potential. Iron reducers, e.g., *Geobacter uraniireducens*, also reduce U(VI) as the electron acceptor through anaerobic respiration. If reducing conditions are sustained, UO_2_ is subject to oxidation by the microbiome. Organic materials such as activated sludge have been tested to sustain reduced conditions, but zero-valent iron in trenches becomes surface-passivated with time due to iron corrosion products and accumulation of biomass, organic material, and precipitates, and its longevity becomes problematic (Kornilovych et al., 2018). Injection of hydroxyapatite [Ca_10_(PO_4_)_6_(OH)_2_] enhances the treatment process because of uranium adsorption on the mineral. Hydroxyapatite releases phosphate with time, yielding insoluble uranium phosphate complexes (Li and Liu, 2022; Lawrinenko et al., 2023). Zhang et al. (2017) simulated the biological aspects of reactive barrier treatment in anaerobic serum bottle microcosms using anaerobic granular sludge as the inoculum. Uranium, added as uranyl sulfate, fractionated into a reduced UO_2_ phase, a soluble phase as uranyl–carbonate complexes, and a sequestered phase in the EPS of the sludge.

Fluctuations in redoxcline depth of sediments impact the sorption and speciation of U(IV) and U(VI) but are weather-, depth-, and site-specific and contingent also on the presence of other reduced compounds such as iron sulfides, organic matter, and contamination with other redox-active metal species. In aquifer sediment redoxclines, Fe(II) compounds are also oxidized biologically and abiotically. The resulting Fe(III) oxides and oxyhydroxides are effective sorptive scavengers of U(VI) species, limiting the ingress of uranyl species into the aquifer. Vanadium oxide (V_2_O_5_) in acid solutions dissolves and forms aggregates through a series of reactions with U(VI), precipitating as crystallized uvanite (U_2_V_6_O_21_·15H_2_O) (Su et al., 2026). Other pathways are also possible, for example, in the presence of K^+^ and Ca^2+^, to form insoluble precipitates. Microbes have no direct role in these phase transitions.

Microbiological oxidation and reduction of uranium species are intimately associated with abiotic redox reactions affecting the stability and mobility of uranium. In wetland uranium-sink sediments, the reduced U(IV) is aggregated with phosphates, Fe(II), and clays, forming non-crystallized Al–P–Fe–Si phases and is sequestered with organic matter in the pore water (Wang et al., 2013, 2014). Sulfate reduction is pervasive in wetlands and forms Fe(II)-sulfide phases, which can sequester Fe(II) from complex phases formed with U(IV) and organic matter. The non-crystallized U(IV) complexes can form colloidal phases that are mobile and can transit in water. U(IV) in these colloidal complexes can be biologically oxidized to ${\text{UO}}_{2}^{2+}$. In addition to the biological reduction of uranium, mineral phases such as pyrite (FeS_2_), pyrrhotite (Fe_n_S_n+1_, n < 1), mackinawite (FeS), structural Fe(II) in clays, and vivianite [${\text{Fe}}_{3}^{2+}$(PO_4_)_2_·8H_2_O] are examples of reduced phases found especially in wetland systems that participate as uranium reductants in the redox phase transformations (Bargar et al., 2013). Some sulfate reducers can use ${\text{UO}}_{2}^{2+}$ as the electron acceptor in anaerobic respiration, but the reduction can also be abiotic by biogenic sulfides produced by sulfate reducers. Kinetically, the bacterial reduction predominates over the abiotic mode of U(IV)O_2_ formation. Brooks et al. (2003) noted that the enzymatic reduction of U(VI) in the Ca–U(VI)–CO_3_ complex formation [Ca_2_UO_2_(CO_3_)_3_] by iron and sulfate reducers was inhibited by calcium ion. This was attributed to the energetically unfavorable reduction of the uranyl electron acceptor in the Ca_2_UO_2_(CO_3_)_3_ complex rather than to chemical interactions of calcium ions with electron donors (lactate, fumarate) or cellular constituents (Brooks et al., 2003).

Several research groups have screened microbial communities in uranium-contaminated environments, such as uranium mine sites. Xia et al. (2020) emphasized the potential of natural microbes at a uranium deposit (Xiangshan, China) to dissolve uranium *in situ*. Mineral-specific attachment of microbes on rock surfaces was noted. Chen et al. (2025) reported microbial community composition data for samples (170–18,000 mg U kg^−1^ soil) collected at four uranium mine sites in China. Not surprisingly, uranium concentration influenced microbial composition; other statistically significant factors included soil pH, total N, Fe(III), and SiO_2_ content. At the Savannah River Site (SC, United States) of the U.S. Department of Energy (http://www.energy.gov/srs/savannah-river-site), soil U–Ni contamination selected for resistance determinants in microbial communities (Pathak et al., 2017; Chukwujindu et al., 2026).

*Exiguobacterium profundum, Pseudomonas putida*, and *Bacillus marisflavi*, isolated from marine sediments, were tested for biofilm formation and capacity of uranium sequestration. FTIR analysis of their biofilms revealed the involvement of amide [–C(=O)N=], carboxyl (–COOH), and phosphate (${\text{PO}}_{4}^{3-}$) functional groups in uranium biosorption (Manobala et al., 2019). Suzuki and Banfield (2004) tested uranium sequestration in *Arthrobacter ilicis* and *Deionococcus radiodurans* isolates retrieved from an acidic, uranium-contaminated site. *A. ilicis* precipitated uranium phosphate intracellularly in close association with polyphosphate granules. *D. radiodurans* precipitated extracellular nanocrystals of uranyl phosphate due to the release of phosphate upon cell lysis. Acid phosphatase (PhoN)-mediated uranium precipitation was also observed in radiation-resistant *D. radiodurans*, whereby PhoN was induced by the radiation-inducible Pssb promoter (Misra et al., 2014). Whether radiation tolerance is an enabling or circumstantial factor in potential *Deinococcus* bioremediation applications remains unclear.

Bencheikh-Latmani et al. (2003) contacted *P. fluorescens* cells with a mixture of uranyl nitrate, citrate, and goethite (α-FeOOH). Almost 99% of the uranium was initially sorbed on the goethite phase before contact with cells. Cell death and lysis released phosphate, which precipitated with uranyl ion as autunite [Ca(UO_2_)_2_(PO_4_)_2_·10-12H_2_O]. Three-month experiments with *P. fluorescens* confirmed the formation of autunite due to phosphate release during cell lysis (Bencheikh-Latmani et al., 2003). Citrate had no specific role in uranium sequestration.

In soil environments, plants can store phosphate as phytic acid, which liberates phosphate for plant growth by the action of phytase and thus may contribute to uranium phosphate precipitation and mineralization (Zhang et al., 2020). Zhang et al. (2026) demonstrated an example whereby a *Stenotrophomonas* isolate provided protection for *Solanum nigrum* Linn. (blackberry nightshade, a traditional herbal medicinal plant) plant growth in a uranium-contaminated environment. Inoculation of *Stenotrophomonas* cells in pot (2 kg soil) experiments increased enzyme activities (urease, acid phosphatase, and catalase) and organic acid concentrations in the plant rhizosphere, thereby promoting the mobility and bioavailability of uranium species. Plant tissue analysis showed that uranium was associated with vacuoles and the cell wall, while plant growth was enhanced as the uranium was detoxified with this immobilization. The rhizosphere uranium concentration decreased by up to 40% in the inoculated plant pots. Organic acid production and enzyme activities over the time course were attributed to *Stenotrophomonas* cells inoculated in the rhizosphere. Several other plant biochemical and rhizosphere changes were observed in these experiments (Zhang et al., 2026).

Qi et al. (2019) also showed in pot (2.5 kg soil) experiments that rhizosphere inoculation (five *Bacillus* spp. and a *Citrobacter* sp.) promoted grass growth in soils containing graded amounts of uranium as uranyl acetate. *Lolium multiflorum* Lam. (annual ryegrass), *Lolium perenne* L. (perennial ryegrass), and *Dactylis glomerata* L. (perennial C3 tufted grass) were grown in pots for 60 days post-inoculation with five different combinations of the bacterial inocula. Uranium content in the stalks and roots increased with the uranium concentration added in the soil (20, 50, 100, and 150 mg U kg^−1^ soil). *Lolium perenne* accumulated more uranium than the other two plant species, almost up to 800 mg U kg^−1^ dry weight. In addition to different responses to uranium among the three plant species, the various combinations of inoculants also resulted in differences in uranium accumulation in plant tissue. Qi et al. (2019) did not elaborate on factors contributing to differences in biological interactions between plant tissue, inoculant combination, and uranyl acetate. The data showed, however, that plant growth-promoting bacteria such as *Bacillus* spp. have a protective role in plant responses to toxic uranium.

Lai et al. (2026) characterized two dicotyledonous sweet potato (*Ipomoea batatas* L.) cultivars in their responses to uranium in soil. The planted pots (10 kg soil) received 100 ± 15 mg U kg^−1^ soil. Over the 120 days of plant growth, the two cultivars differed in uranium accumulation in various sections of plant tissues. Uranium induced plant hormone metabolism, and the plants produced secondary metabolites, including cyclic nucleotides (Lai et al., 2020, 2021). Soil microbiome responses included changes in the dominant phyla and a reduction in bacterial diversity, and the responses to uranium and cadmium were also apparent in changes in rhizosphere metabolites. In general, plant rhizosphere-microbiome studies have shown strong interactions and primary and secondary metabolic responses to uranium in soil, all reflected in plant growth, exudates from roots, and changes in microbes and metabolites in the rhizosphere. Induction of antioxidants and other metabolic pathways of detoxification in plants and rhizosphere microbes are typical responses to uranium stress conditions (Zhu et al., 2026). Soil phosphate levels are an important parameter in the rhizosphere because of their precipitation as biologically unavailable uranium phosphates (Yuan et al., 2025).

Zhang et al. (2025) used biochar loaded with various combinations of *Bacillus* and *Pseudomonas* cells to treat U(VI)-contaminated soil (50 mg U kg^−1^) in pot (100 g soil) experiments. Uranium content in the experiment was analyzed with DTPA (diethylenetriamine penta-acetic acid) extraction, which recovers the soluble uranium fraction. After 80 days, the DPTA-extractable fraction of uranium decreased by 80% due to uranium immobilization by reduction, adsorption, and precipitation. U(VI) was not reduced in the abiotic control. Spectral analysis indicated the presence of amino, hydroxyl, carboxyl, and phosphate groups associated with inoculated biochar.

*A. ilicis* sequestered uranium in intracellular precipitates closely associated with volutin-like polyphosphate granules. This type of intracellular sequestration may be interpreted as a uranium detoxification mechanism; however, it should be noted that uranyl ions have a high affinity for many other cellular molecules, including nucleotides and proteins, which may compromise cell metabolism and replication. In *Kocuria* cultures, the cell surface provided nucleation sites for uranium precipitation Wang et al., (2019). SEM–EDS results showed elevated levels of P and U in the precipitates on the cell surface, and XPS analysis confirmed the presence of P–O–U bonds. (Wang et al. 2019) noted that FTIR results showed the presence of C=O, –OH, and –COOH functional groups in uranium complexation in addition to phosphates.

*Pantoea* sp. TW18, isolated from radionuclide-contaminated soils, accumulated 77 mg U g^−1^ dry wt at pH 4.1 through complexation with carboxyl, amino, and phosphoryl functional groups on the cell surface (Zhang Z. et al., 2018). Based on FTIR and XPS spectra, nitrogen- and oxygen-containing functional groups formed the primary uranium complexes on the cell surface. Incubation of uranium tailings (320–1820 mg kg^−1^) with a *Pantoea* and *Desulfovibrio* combination yielded multiple stable products (Lv et al., 2022). Sulfate reducers reduced uranium in the tailings sample to UO_2_ and precipitated uranium phosphate on the cell surface; *Pantoea* dissolved uranium from the tailings sample and precipitated it mostly as uranium phosphate on the cell surface.

Morcillo et al. (2014) showed that uranium complex formation in *Idiomarina loihiensis* MAH1 (a marine species) was pH-dependent and was affected by the chemical solute composition. Uranium complex formation was predominant with organic phosphate groups at pH 2, and phosphate and carboxyl groups at pH 3 and 4.3. The uranyl phosphate phase precipitated on the cell surface resembled meta-autunite.

Macaskie et al. (2000) reported the accumulation of uranium by a *Citrobacter* isolate as uranyl phosphate on the cell surface. The precipitation occurred on the LPS layer, a structural component of the cell wall, which contains monophosphate groups of the lipid A backbone. A phosphatase action was proposed that would release phosphate from LPS and other P sources on cell surface components for uranium phosphate formation. In the *Citrobacter* study, acid phosphatase was present in the outer membrane as well as in cell exudates, which suggested an enzymatic mechanism for releasing phosphate from LPS for uranium complexation (Macaskie et al., 2000). Xie et al. (2008) worked out the kinetics of uranium biosorption in *Citrobacter freudii* (*t* = 180 min) and concluded that carboxyl groups in the cell wall were the important sorption sites. Zeng et al. (2022) suggested the induction of intracellular formation of uranium phosphate complexes by an alkaline phosphatase; however, the biochemical and genetic aspects of induction and expression of phosphatases are unresolved in uranium phosphate complex formation.

Merroun et al. (2006) isolated *Microbacterium oxydans* and a *Sphingomonas* sp. from a subsurface monitoring well site at the radioactive subsurface repository in Siberia. Resting cell suspensions were contacted with 0.5 mM uranyl nitrate solution at pH 2.0 and 4.5 for 48 h. X-ray absorption fine-structure spectroscopy showed the precipitation of autunite at pH 4.5 in both test cultures. At pH 2.0, uranium also formed complexes with organophosphate groups on the cell surface. More uranium was sequestered at pH 4.5 than at pH 2.0. TEM observations indicated that uranium accumulated on the cell surface of *M. oxydans*. In contrast, the *Sphingomonas* isolate contained uranium in the cell membrane and intracellular electron-dense granules.

Barkleit et al. (2008, 2011) characterized complex formation between uranyl ion and LPS from *P. aeruginosa* over a wide range of pH and concentrations. Uranyl ion formed coordination complexes with both carboxyl (–COOH) and phosphoryl (–${\text{PO}}_{3}^{-}$) groups. Uranyl–phosphoryl coordination dominated at excess LPS, but carboxyl groups were increasingly important at low LPS concentrations. At neutral pH, uranyl ion was retained on the surface of LPS through chelation with carboxyl and hydroxyl groups located in the outer core (Lins et al., 2008).

## 6 Conclusion

Many physical and chemical factors are comparable in the heterotrophic bioleaching and acid ferric-iron bioleaching of uranium from ores and rocks, such as particle size, pulp density, contact time, chemical composition and mineralogy of the ore sample, acid demand, secondary dissolution products, and solid-phase transformations. The types and concentrations of carboxylic acids produced by heterotrophic bacteria directly impact the dissolution of uranium during contact with the solid phase in the pulp. Heterotrophic bacterial leaching processes are not considered viable for commercialization. Although heterotrophic biomass yields are orders of magnitude higher than those of the acidophilic Fe^2+^-oxidizing bacteria used in acid ferric sulfate bioleaching, their growth media are not selective, and potential contamination during biomass production and the contact of spent media with the pulp are practical issues of concern.

The redox state of uranium is subject to biological reduction and oxidation. The reduction of hexavalent uranium produces solid-phase UO_2_, which does not traverse biological membranes, although it may form deposits on the cell envelope. The oxidation of tetravalent uranium mobilizes it to the bioavailable form ${\text{UO}}_{2}^{2+}$, unless it is sequestered with functional groups on the cell wall. Phosphates are common ligands for uranium phosphate precipitation, and some form crystallized phases. Uranyl phosphate phases are neither soluble nor bioavailable, and their formation alleviates the inhibitory effect of uranium on microbes. Bacterial siderophores, EPS, and LPS have functional ligands to sequester uranium. Experimental data on uranium sequestration by heterotrophic bacteria reported in the past 25 years are varied, but a common trend with cell responses and sequestration is apparent. The various solid phases of uranium, particularly phosphates, may be a potential approach for remediation, but their biological and chemical properties and stability are inadequately characterized. In contaminated soils, the decrease in bioavailable uranium by reductive formation of UO_2_ or by biomineralization treatment facilitates the evolution and establishment of microbial populations, which can further sequester residual bioavailable uranium and help stabilize the community structure.

Heterotrophic bacteria may have application in the sequestration of uranium, and possibly other actinides, in specific cleanup or bioremediation situations involving uranium-containing spills or residual hazardous radioactive or actinide waste. Such applications would warrant targeted research to identify the best combinations of organisms and growth conditions to maximize biomass growth and the cellular fractions responsible for sequestration. Bioremediation applications to immobilize uranium as UO_2_ are promising, but success also depends on preventing oxidation of UO_2_ to soluble species. Wetland systems involve vertical biogeochemical redox gradients of uranium that control oxidation and reduction processes. Organic compounds with silicates from clays, and Al, Fe, and Mn, in wetlands form colloidal uranium complexes that may protect uranium from reduction and precipitation. It is the biologically mediated redox transitions and precipitation with functional ligand groups of bacteria, and also ligands in fungal biomass, that may be key to developing biological approaches and techniques to abate and treat uranium pollution in contaminated environments.

Neither the premise nor the application of using bacteria to dissolve, capture, and sequester uranium from various sources, including contaminated aquifers, has matured into a treatment process. Individual studies have shown several examples and biological mechanisms of uranium sequestration and redox changes, but the integration of empirical and experimental information and process modeling remains in its infancy. Multiple and concurrent biological mechanisms of uranium redox changes and biogenic sequestration in dynamic environments are key challenges for thermodynamic analysis and modeling. Multi-scale parameters relevant in environmental scenarios, derived from biological mechanisms of uranium dissolution and sequestration, have not been critically evaluated to assess their applicability and the theoretical basis linking uranium speciation with chemical, physical, and biological recovery from uranium-contaminated solid and solution phases. Adding to the research challenges, microbial diversity, interactions, kinetics, and decay as influenced by uranium under selective conditions are poorly predictable and not well understood beyond well-defined bacterial cultures in the laboratory. The complex factors that govern *in situ* and *ex situ* uranium bioprocesses and microbial population dynamics require multidisciplinary, targeted research and development.
